# Polymerase I and Transcript Release Factor Acts As an Essential Modulator of Glioblastoma Chemoresistance

**DOI:** 10.1371/journal.pone.0093439

**Published:** 2014-04-18

**Authors:** Xin Wang, Tianzhu Liu, Yifeng Bai, Hongzhan Liao, Shengcong Qiu, Zhenhua Chang, Yanting Liu, Xiaohui Yan, Hongbo Guo

**Affiliations:** 1 The National Key Clinic Specialty, the Neurosurgery Institute of Guangdong Province, Guangdong Provincial Key Laboratory on Brain Function Repair and Regeneration, Department of Neurosurgery, Zhujiang Hospital, Southern Medical University, Guangzhou, P. R. China; 2 Department of Neurosurgery and Institute for Functional Brain Disorders, Tangdu Hospital, The Fourth Military Medical University, Xi'an, P. R. China; 3 Department of Oncology, Sichuan Academy of Medical Sciences & Sichuan Provincial People's Hospital, Chengdu, P. R. China; 4 Department of Laboratory Medicine, Tongchuan People's Hospital, Tongchuan, P. R. China; 5 Clinical Research Centre, Nanfang Hospital of Southern Medical University, Guangzhou, P. R. China; Okayama University, Japan

## Abstract

**Objectives:**

This study is to investigate if polymerase I and transcript release factor (PTRF) acts as a modulator in glioblastoma (GBM) chemoresistance.

**Methods:**

Multidrug resistant (MDR) GBM cell line U251AR was established by exposing the U251 cell line to imatinib. The 2D-DIGE and MALDI-TOF/TOF-MS were performed on U251 and U251AR cell lines to screen MDR-related proteins. The expression of PTRF was determined by Western blot and quantitative RT-PCR analyses.

**Results:**

When compared with the parental U251 cells, expression of 21 proteins was significantly altered in U251AR cells. Among the 21 differentially expressed proteins, the expression of PTRF was up-regulated by 2.14 folds in U251AR cells when compared with that in the parental U251 cells. Knockdown of PTRF in GBM cell lines significantly increased chemosensitivity of cells to various chemical drugs and decreased the expression levels of caveolin1, a major structural component of caveolae. Expression levels of PTRF and caveolin1 were significantly up-regulated in the relapsed GBM patients. The mRNA level of PTRF and caveolin1 showed a positive correlation in the same GBM specimens.

**Conclusions:**

Our results indicate that PTRF acts as a modulator in GBM chemoresistance.

## Introduction

Glioblastoma (GBM) is one of the most lethal diseases in the central nervous system of adults and the median survival time of GBM patients is 12 months [1]. There are various therapeutic methods for GBM, including surgery, chemotherapy and radiotherapy. However, the median survival time of patients with GBM was only modestly increased to 15 months [2]. Major limitations of therapies for GBM are tumor recurrence after surgery, tumor infiltration into surrounding tissues, and intrinsic or acquired resistance to chemotherapy and radiotherapy [3].

Although the DNA-methylating agent temozolomide (TMZ) has been developed for treatment of gliomas [2], several growth factor receptors such as PDGFR and EGFR have been used as therapeutic targets [4], [5]. Treatment with the PDGFR/c-KIT/abl kinase inhibitors dramatically inhibited the viability and anchorage-independent growth of tumor cells [6]. But only 10–20% of the patients had a clinical response to these inhibitors, and most of these patients subsequently exhibited rapid tumor progression due to drug resistance [7]. Wilson et al [8] found that inhibition of RTK ligands could reverse both innate and acquired resistance. However, the mechanisms underlying the resistance to RTK inhibitors have not yet been fully elucidated [9].

Imatinib is one of the representative RTK inhibitors. Antagonism of imatinib in glioma models has been demonstrated to successfully inhibit tumor growth both in *vitro* and in *vivo* [10]. We constructed an imatinib-resistant GBM cell line U251AR in our previous study [11] and used the two-dimensional difference gel electrophoresis (2D-DIGE) and mass spectrometry (MS)-based proteomic approaches to study the chemoresistance-associated proteins in GBM cells. Proteomics is a powerful and effective tool to evaluate protein profiles [12]. 2D-DIGE is a sensitive gel-based method for protein separation and quantification. Proteins are pre-labeled with different fluorescent dyes, mixed, and separated on gels [13]. Proteomics offers the potential ability to find unknown mechanism involved in MDR of cancers and provides new opportunities to find biomarkers and therapeutic targets for tumors [14].

Polymerase I and transcript release factor (PTRF), also known as cavin1, is originally identified as a protein involved in dissociation of transcription complexes in *vitro* [15]. PTRFs in cell surface are associated with processes of vesicular transport, cholesterol homeostasis [16], [17], and lipolysis control [18]. PTRF mutations are associated with congenital generalized lipodystrophy in humans [19]. Interactome analyses suggest that PTRF has unknown functions besides the roles described above [20]. Loss of PTRF expression in prostate cancer and lung cancer has been demonstrated to be related with tumor progression [21], [22]. The caveolae structural proteins of PTRF and caveolin1 are essential for MDR of breast cancer [23]. PTRF induces formation of abundant caveolae in various cultured cells and in zebrafish embryos [24], [25]. PTRF and caveolin1 are closely associated on the plasma membrane [25]. The caveolin proteins have been reported to be located in caveolae and essential for the presence of caveolae [26]. Quann [27] and his colleagues reported that over expression of caveolin1 in the GBM cell line U87 negatively regulated cell growth and survival pathways. Expression of caveolin1 is up-regulated in GBM cell lines and tumors compared to primary human astrocytes and normal brain tissues [28], [29]. The cells resistant to TMZ affect caveolin1 expression in *vitro* and in *vivo* in human GBM models [30]. However, there is no study on expression of PTRF in GBMs. Thus, in this study, we investigated expression and function of PTRF in GBM cell lines and patients. The role PTRF in chemoresistance of GBM cell lines was also analyzed. Our data indicate that PTRF may be used as valuable targets for developing new therapeutic strategies for GBM patients.

## Materials and Methods

### Ethics statement

Prior written and informed consent was obtained from the patients and the guardians on behalf of children enrolled in this study. This study was approved by the ethics review board of Southern Medical University.

### Tissue specimens

Patient specimen samples were obtained from Zhujiang and Nanfang Hospital (Southern Medical University, Guangzhou, China). Patients enrolled in this study included 8 grade I astrocytoma cases, 13 grade II astrocytoma cases, 10 grade III astrocytoma cases, and 27 GBM cases. Among 27 GBM cases, 6 GBM cases were relapsed 6 months after TMZ therapy. All patients gave prior written and informed consent prior to collection of specimens according to institutional guidelines of Southern Medical University. Tissue samples were snap-frozen in the operation room immediately after surgery. Non-tumor tissues were diagnosed by a board-certified neuropathologist. Normal tissues were confirmed to be tissues surrounding tumor and free of cancer cells according to pathologic examination. For each patient, a frozen tumor sample (stored at −80°C) and a paraffin-embedded tissue specimen was available.

### Cell lines and cell culture

Human GBM cell line U251 was obtained as a gift from College of Public Health, Southern Medical University, Guangzhou, China [11]. The MDR cell line U251AR was established and maintained in our laboratory. The cells were cultured in Dulbecco's modified Eagle's medium (DMEM/H) containing 10% (v/v) fetal bovine serum (FBS), penicillin (200 U/mL) and streptomycin (100 µg/mL). Cells were cultured at 37°C in a humidified incubator with an atmosphere of 5% CO_2_. The U251AR cell line was established by exposing the U251 cell line continuously to increasing concentrations of imatinib (STI571) over a period of 12 months in our lab. To maintain the MDR phenotype of U251AR cells, imatinib was added to the medium at a final concentration of 122 µg/mL during U251AR cell culture.

### Immunofluorescence

A total of 3×10^5^ cells per chamber were placed into Lab-Tek two-chamber slides and incubated overnight. On the next day, when cells were 50–70% confluent, they were washed with PBS twice, fixed in 4% paraformaldehyde (Sigma, St. Louis, Missouri, USA) and permeabilized in 0.1% Triton X-100 (Sigma, St. Louis, Missouri, USA) at 4°C for 30 min. The cells were then washed 3 times with PBS and incubated with blocking solution (10% horse serum in PBS). After blocking, cells were incubated with primary antibodies against PTRF or caveolin1 overnight at 4°C. After washing with PBS for three times, cells were incubated with the secondary antibody of goat anti-rabbit-Alexa Fluor 488 (1∶1,000; Molecular Probes, Invitrogen, USA) for 1 h at room temperature in the dark. Finally, the cells were washed three times with PBS and incubated with 0.25 mg/ml DAPI (Roche, Mannheim, Germany) for 1 min at room temperature in the dark. After extensively washing with PBS, samples were imaged on a confocal laser scanning microscope (Olympus Fluoview, Tokyo, Japan) using a 60× oil immersion objective, with identical exposure times.

### Protein extraction

Cell lysates were prepared from U251 and U251AR cell lines by mechanical disruption in ice-cold lysis buffer (Tris 20 mM, pH 7.5, CHAPS 4%, urea 8 M (Sigma, St Louis, USA)) and antiproteases cocktail (Complete EDTA-free tablets, Roche Diagnostics, Mannheim, Germany). Samples were sonicated (6 cycles of ten seconds with relapse of 30 seconds in ice-bath) and centrifuged (15000 g, 30 minutes, 4°C). Supernatants were ultracentrifuged at 108,000 g for 60 minutes at 4°C. Protein concentration was determined using the Bradford protein assay and the extracted protein (100 µg) was kept at −80°C.

### Protein labeling with cyanin dyes

Cytosolic extracts were labeled with CyDyes DIGE Fluors (GE Healthcare, Bucks, UK) according to the manufacturer's recommended protocol. Briefly, 50 µg of each sample were minimally labeled with 400 pmol amine-reactive cyanine dyes, Cy3 or Cy5, on ice for 30 minutes, in the dark. U251 and U251AR were all labeled with Cy5 or Cy3 for different gels. An internal pool, labeled with Cy2 fluorescent dye, was generated by combining equal amounts of U251 and U251AR cells together. The labeling reaction was quenched by incubation with 1 µL of 10 mM lysine (Sigma-Aldrich, ST Louis, USA) on ice in the dark for 10 minutes. Following the labeling reaction, the U251 cell extracts and the U251AR cell extracts were combined together with the internal pool, and Destreak™ IEF buffer (GE Healthcare) was added to make the volume up to 450 µl prior to IEF (isoelectric focalisation) on five 24 cm gel strips.

### Two-dimensional SDS-PAGE

The isoelectric electrophoresis was carried out using an IPGphor™ system (GE Healthcare). Pre-cast immobilized pH gradient strips (pH 3–10 NL, 24 cm) were used for the one-dimensional separation with a total focusing time of 60 kV-h. After IEF, the IPG strips were incubated two times at ambient temperature for 15 minutes in an equilibration solution (0.05 M Tris-HCl pH 8.8, 6 M Urea, 30% glycerol, 2% SDS and bromophenol blue) containing 65 mM DTT and 250 mM iodoacetamide. Strips were directly applied on top of pre-cast 12% SDS-PAGE gels (GE Healthcare) and ran in a vertical Ettan DaltSix system (GE Healthcare) for approximately 5 hours. Another gel ran in the same way for picking of protein pots. Four gels were processed simultaneously.

### Gel imaging and data analysis

After SDS-PAGE, cyanine-labeled proteins were directly visualized using a Typhoon™ 9400 imager (GE Healthcare) in a fluorescence mode. Cy2, Cy3 and Cy5 images were scanned using 488 nm, 532 nm, and 633 nm laser, respectively. Each gel was scanned at 200 µm (pixel size) resolution and was processed using the DeCyder software V5.01 (GE Healthcare), followed by quantification, gel matching and statistical analyses. To exclude artifacts from gel images and differentially quantify the protein spots in the images, the Differential In-gel Analysis module (DIA) was used for pair-wise comparison of the two samples (U251 and U251AR) on each gel. The Biological Variation Analysis module (BVA) was used to match the entire set of protein-spot maps from comparable gels simultaneously. Student's test (p<0.05) was performed for statistical analyses. Protein spots with at least 1.5-fold changes in volume after normalization were defined as differentially regulated. The statistical power of the analysis was calculated similarly to results reported by Engelen K. et al and [31] Karp N. et al [32]. The standard deviation of the log10 (standardized abundance) per condition was calculated for each spot that have been matched across the 2 gels of the analysis. The median of these standard deviations was calculated in each condition to estimate the global variance of the replicates. After 2D-DIGE imaging and analysis, another gel was stained with Coomassie-blue. Gels were scanned (Image Scanner TM GE Healthcare) and stored in 1% acetic acid at 4°C until spot excision. Matching between Coomassie-blue stained gels and fluorescence maps was performed manually and the pick lists were generated using the Image Master™ 2D Elite software (GE Healthcare).

### MALDI-TOF/TOF mass spectrometry

Coomassie Blue-stained protein spots were excised from 2-D gels and processed using an Ettan™ Spot Handling Workstation (GE Healthcare). Gel plugs were washed 3 times in MilliQ water, followed by a rinse in 50% methanol/50 mM ammonium bicarbonate and a rinse in 75% ACN to ensure complete removal of dye and detergent. After drying, gel pieces were re-hydrated for 60 minutes in 20 mM NH_4_HCO_3_ with 16.6 µg/ml porcine trypsin (Promega, Charbonnières-lesbains, France). Extraction was performed in two successive steps by addition of 50% ACN and 0.1% TFA, respectively. The digestion products were dried out and dissolved in 2 mg/mL a-cyano-4-hydroxycinnamic acid in 70% ACN/0.1% TFA, before spotting onto MALDI targets (600 µm 384 Scout MTP AnchorChip™; Bruker Daltonics, GmbH, Bremen, Germany). Peptide mass fingerprints were obtained using a MALDI-TOF/TOF mass spectrometer (Ultraflex™; Bruker Daltonics, GmbH) and processed using the FlexAnalysis™ software (version 2.2; Bruker Daltonics, GmbH) for generation of peak list and an internal calibration with trypsin auto-digestion peptides. Peak lists were then transferred to ProteinScape™ software (version 1.3; Bruker Daltonics, GmbH) for another automatic calibration based on a calibration list (related to the sample type and treatment) containing autolysis peaks and contaminants (keratins, polymers and background peaks). After re-calibration, an automatic trypsin and contaminants filtering and removal were performed in order to get the m/z ratio and to obtain high identification rates (Score-Booster). Only the monoisotopic masses of tryptic peptides were then used to query NCBInr sequence databases using the Mascot search algorithm (Mascot server version 2.1.04; http://www.matrixscience.com). Search conditions were as follows: an initial mass window of 70 ppm for the internal calibration, only one missed cleavage acceptable, modification of cysteines by iodoacetamide and methionine oxidation as variable modifications. Results were scored using the probability-based Mowse score (the protein score is −10× log (P)). P is the probability that the observed match is a random event. In our experiment, a score greater than 90 was considered as a significant identification (p<0.05).

### Immunoblot analyses

Cytosolic protein extracts (10–30 µg) were loaded on 12% polyacrylamide gels for performing 1D-SDS-PAGE. The biotinylated ECL western blotting molecular weight markers (Amersham-GE-Healthcare) were used. The proteins were transferred onto PVDF membranes (Millipore, USA). Equal amount of proteins that were obtained from U251, U251AR and other cells and quantified by Bradford protein assay were loaded on each gel. Non-specific sites were blocked in Tris-buffered saline (TBS) containing 5% (w/v) non-fat dry milk and blots were incubated with diluted primary antibodies in 0.1% Tween 20 and 1% nonfat dry milk TBS. The primary antibodies included rabbit monoclonal anti-human PTRF (dilution 1∶1000, Abcam, Cambridge, Massachusetts, USA), rabbit monoclonal anti-human caveolin1 (dilution 1∶1500, Cell Signaling Technology, Danvers, Massachusetts, USA), rabbit monoclonal anti-human VIM (dilution 1∶1000, Cell Signaling Technology, Danvers, Massachusetts, USA) and goat monoclonal anti-human P-gp (dilution 1∶200, Santa Cruz Biotechnology, Santa Cruz, California, USA). β-actin was used as an internal control. After washing in TBS, blots were incubated with secondary antibodies of peroxidase-conjugated IgG (dilution 1∶5000, Santa Cruz, California, USA) and Streptavidin-HRP (for biotinylated markers). The enhanced chemiluminescence system ECL+ (GE Healthcare) was used for color development.

### Reverse transcription-quantitative PCR

Total RNA was extracted using Trizol reagent (Invitrogen, USA). Total RNA was reversely transcribed using prime Script RT reagent Kit (Takala, Dalian, China). Quantitative RT-PCR was carried out in an MX7500 sequence detection system (Stratagene, USA) using SYBR Green according to the manufacturer's instructions. Primers were listed in Table 1. Glyceraldehyde 3-phosphate dehydrogenase (GAPDH) was used as an internal control. All samples were normalized to internal controls and fold changes were calculated through relative quantification (2ΔΔCT).

**Table 1:** Primer sequences used in quantitative RT-PCR analysis.

Genes	Primers
PTRF	Forward: ACGCCACCACGAGCAATAC
	Reverse: CTCCGACTCTTTCAGCGATTT
CAV1	Forward: AGAACCAGAAGGGACACACAGT
	Reverse: AGATGGAATAGACACGGCTGAT
VIM	Forward: GTTTCCAAGCCTGACCTCAC
	Reverse: GCTTCAACGGCAAAGTTCTC
P-gp	Forward: CCCATCATTGCAATAGCAGG
	Reverse: TGTTCAAACTTCTGCTCCTGA
MRP1	Forward: ATGTCACGTGGAATACCAGC
	Reverse: GAAGACTGAACTCCCTTCCT
BCRP	Forward: ATGTCACGTGGAATACCAGC
	Reverse: GAAGACTGAACTCCCTTCCT
GAPDH	Forward: GAGGTGATAGCATTGCTTTCG
	Reverse: CAAGTCAGTGTACAGGTAAGC

### PTRF knockdown

BLOCK-iT Pol II miR RNAi expression vector kit (Invitrogen Co., Carlsbad, California, USA) was used to induce knockdown of PTRF. Briefly, single-stranded miRNAs were annealed to form double-strands, and inserted into the pcDNA6.2-GW/EmGFP-miR vector (Invitrogen, USA). Short hairpin RNA (shRNA) targeting PTRF was named as shPTRF. The empty vector was named as shNC. The nucleotide sequences of the target miRNA and unrelated miRNA were shown in Table 2. The plasmids were transfected into U251AR and U251 cells by Lipofectamine™ 2000 (Invitrogen, USA) according to the manufacturer's instructions. After incubation for 24 h, 500 ng/mL Blasticidin S HCl (Invitrogen, USA) was added into medium. After transfection, PTRF mRNA level was determined by quantitative RT-PCR. Subsequently, several clones with lower expression levels of PTRF mRNA were analyzed further for their PTRF protein levels by Western blotting. Finally, clones with effective PTRF knockdown were selected for further analyses. Cells transfected with an empty vector were used as a control.

**Table 2:** The nucleotide sequences of the target miRNA and the unrelated miRNA.

Target miRNA	Sense: TGCTGTGTTCATGCGCTTCTCCAGGGGTTTTGGCCACTGACTGACCCCTGGAGGCGCATGAACA
	Antisense: CCTGTGTTCATGCGCCTCCAGGGGTCAGTCAGTGGCCAAAACCCCTGGAGAAGCGCATGAACAC
Unrelated miRNA	Sense: TGCTGAAATGTACTGCGCGTGGAGACGTTTTGGCCACTGACTGACGTCTCCACGCAGTACATTT
	Antisense: CCTGAAATGTACTGCGTGGAGACGTCAGTCAGTGGCCAAAACGTCTCCACGCGCAGTACATTTC

### *In vitro* drug sensitivity assay

Cells were placed in 96-well plates at a density of 2×10^3^ per well in a final volume of 100 µL and transfected with shNC and shPTRF. Cell viability was analyzed after incubation with 100 µg/mL TMZ for 24 h, 48 h, 72 h, 96 h and 120 h. The cell viability assay was performed using a CCK8 kit (Dojindo Molecular Technologies, Japan). In drug sensitivity analysis, cells were reseeded in 96-well plates 24 h post-transfection with a density of 1.5×10^4^ per well and treated with imatinib, VP-16, or TMZ (50 to 200 µg/mL) for 48 h.

### Immunohistochemical analysis

Expression levels of PTRF and caveolin1 in tissues were detected by an ultrasensitive S-P kit (Zhongshan Biotechnology Co. Ltd, Beijing, China) according to the manufacturer's recommendation. Rabbit monoclonal primary antibodies against human PTRF (dilution, 1∶100; Abcam, Cambridge, Massachusetts, USA) and caveolin1 (dilution, 1∶150; Cell Signaling Technology, Danvers, Massachusetts, USA) were used. Polyperoxidase rabbit IgG was used as the secondary antibody (Zhongshan Biotechnology Co. Ltd, Beijing, China). Sections were analyzed with bright field microscopy (Olympus BX51, Tokyo, Japan). Negative controls were also detected with the primary antibody. Immunostained sections were examined by light microscopy using ×40 objective lens and ×10 eyepieces. Immunostaining intensity (IS) was counted by the Image pro-Plus 6.0 software.

### Statistical analysis

All experiments were performed in triplicate. The results were given as means ± standard deviations (SDs). Statistical analyses were performed using either an analysis of variance (ANOVA) or Student's t test. The relationship between the PTRF and Caveolin-1 mRNA levels in the same GBM specimens were investigated by Pearson correlation. The difference was considered statistically significant when the P value was less than 0.05. All statistical analyses were carried out with SPSS 13.0 software.

## Results

### Imatinib-resistant GBM cell line U251AR was established successfully

**Figure 1:**
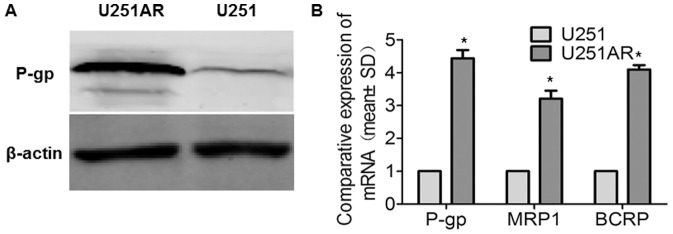
The biological characteristics of MDR cell line U251AR. (A) The expression of P-gp was significantly increased in chemoresistant cell line U251AR as detected by Western blot analyses. (B) The mRNA levels of P-gp, MRP1, and BCRP in U251 and U251AR cells were determined by quantitative RT-PCR. Values of three independent experiments were represented as the mean ± SD. *, P<0.05.

By using the parental cell line U251, we previously established the imatinib-resistant GBM cell line U251AR, which had a cross-resistance to VP-16 and TMZ. The U251AR was cultured in medium with imatinib (122 µg/mL) to maintain the MDR phenotype. The MDR phenotypes of imatinib-resistant cell line U251AR included up-regulation of some cellular genes. In this study, we tested the ATP-dependent drug efflux pump (P-gp) expression by Western blotting and the mRNA levels of P-gp, MRP1 and BCRP by quantitative RT-PCR in U251AR in comparison with the parental cell line U251. The P-gp, MRP1 and BCRP were significantly increased in drug-resistant cell line U251AR (*, P<0.05) (Fig. 1). These results suggest that the imatinib-resistant GBM cell line U251AR was established successfully.

### Proteome profiling of U251 and U251AR cell lines

**Figure 2:**
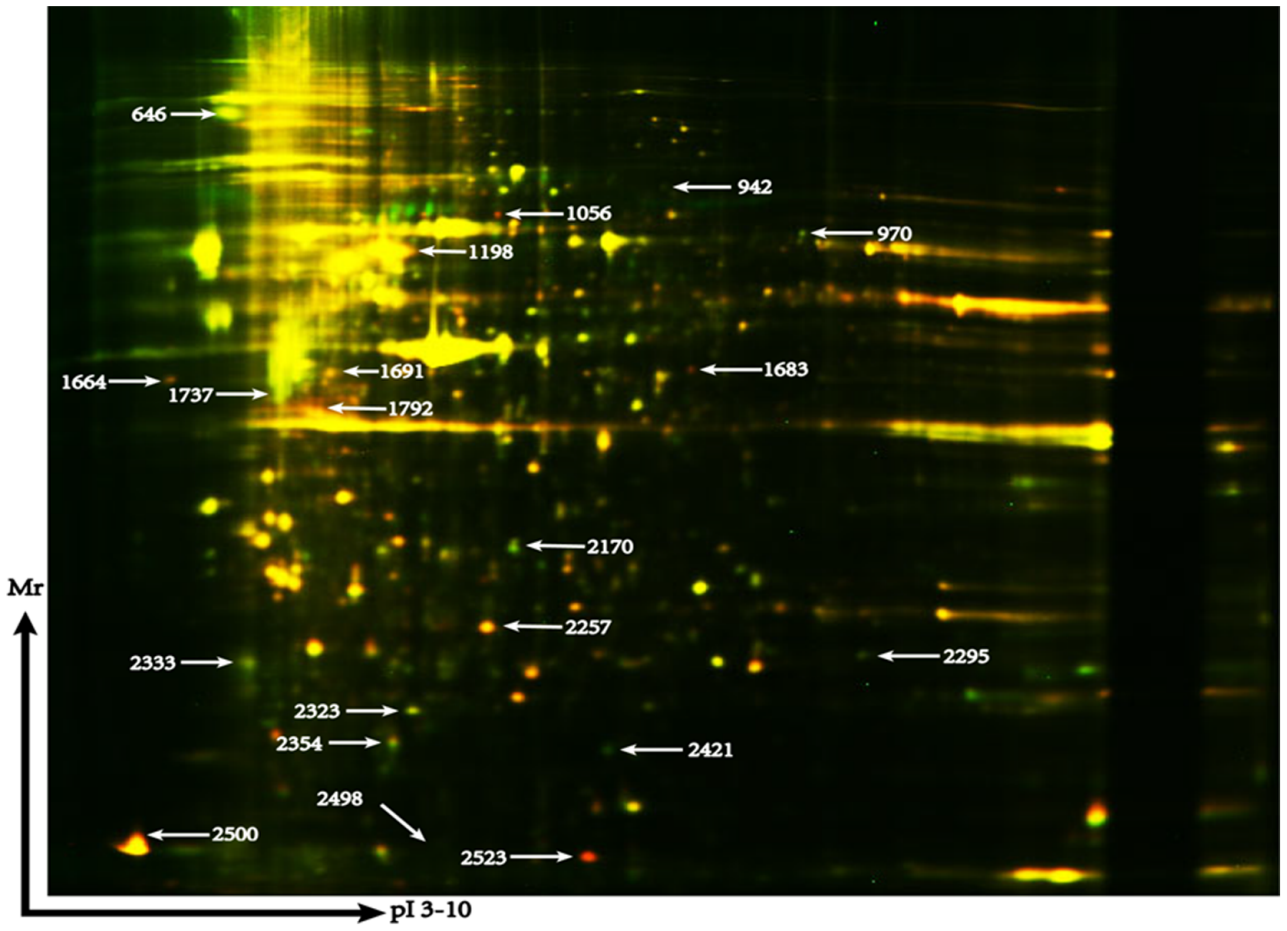
Proteomic analysis of GBM cells by 2D-DIGE. A representative 2D-DIGE image (merged image) showed the protein profile of U251AR and U251 cells, labeled with Cy3 (green spots) and Cy5 (red spots), respectively. The approximate molecular weight range in the vertical dimension was from 10 to 150 kD. The PI of proteins ranged from 3 to 10. The differently expressed protein spot ID were indicated with white arrows. The protein spot 2421 and 1737 was PTRF and VIM, respectively.

**Figure 3:**
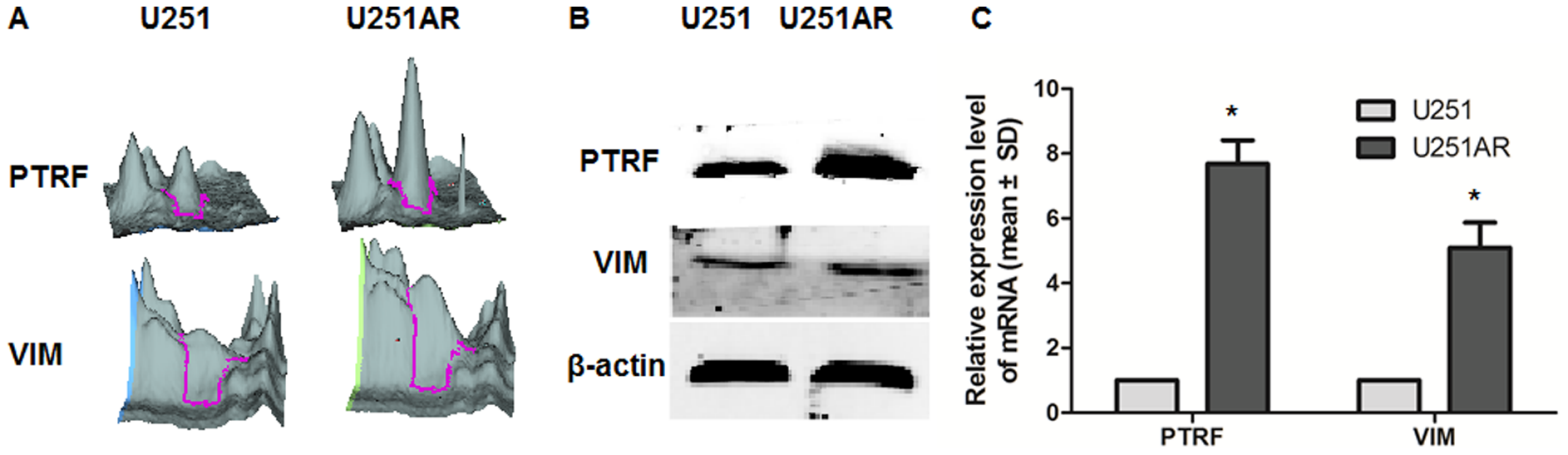
The expression of PTRF and VIM in U251AR and U251 GBM cells. (A) 3-D view of PTRF and VIM proteins showed their expression in U251 and U251AR cells. (B) Western blotting and (C) RT-PCR results indicating the expression of PTRF and VIM in U521AR and U251 cells.

To obtain a global protein image of U251 and U251AR cells, we performed three 2D-DIGE gels to detect differently expressed proteins. For each gel, a merged image was generated from three images of the U251, U251AR, and the internal standard samples. A representative DIGE gel with merging of Cy3 and Cy5-labeled images was shown in Fig. 2. A total of 2516 to 2735 spots were detected in the DIA workspaces using DeCyder software. In the BVA module, 41 spots were found to be differentially expressed based on the criteria that an average ratio was more than 1.5 or less than 1.5 (P value<0.05). Among them, 23 spots were found to be down-regulated and 18 spots up-regulated in the chemoresistant U251AR when compared with U251. Some protein spots might be undetectable in gel stained with Coomassie Blue because of their low expression levels. Twenty-one protein spots with high abundance were found with significantly altered expression in both cell lines as indicated by the MALDI-TOF/TOF MS analysis. Among the 21 differentially expressed proteins, 9 proteins were up-regulated and 12 proteins were down-regulated in U251AR cell line (Table 3), including PTRF and VIM. The 3-D view of PTRF and VIM proteins were showed in Figure 3A.

**Table 3:** Twenty-one differentially expressed proteins in U251 cell line versus U251AR cell line.

Master numbers	Accession no	Genes	Protein MW^a^	Protein PI	Pep. Count	Total ion score	Fold changes^b^	Overall trend
646	P14314	PRKCSH	60357.2	4.33	5	188	2.57±0.410	up^c^
942	P05556	TGB1	91664.2	5.27	1	46	1.61±0.170	up
970	Q9UQR0	SCML2	78063.2	8.79	3	183	−1.63±0.169	down^d^
1056	P61978	HNRNPK	51229.5	5.39	2	159	−1.68±0.184	down
1198	P68363	TUBA1B	50803.9	4.94	7	402	−1.67±0.127	down
1218	P07437	TUBB	50095.1	4.78	10	672	−1.67±0.057	down
1664	Q01105	SET	33468.7	4.23	2	153	−1.93±0.212	down
1683	P49116	NR2C2	66228.5	5.89	4	215	−1.85±0.156	down
1691	P08865	RPSA	32947.5	4.79	7	322	1.69±0.113	up
1737	P08670	VIM	53676.1	5.06	9	497	1.57±0.127	up
1792	P06748	NPM1	32725.9	4.64	3	188	−1.63±0.226	down
2170	P40261	NNMT	30011.2	5.56	2	137	−1.65±0.240	down
2257	P09936	UCHL1	25150.6	5.33	6	504	−1.53±0.113	down
2295	P31943	HNRNPH1	49483.5	5.89	4	176	1.54±0.169	up
2323	P07355	ANXA2	38807.9	7.57	3	126	1.57±0.171	up
2333	O00264	PGRMC1	21771.8	4.56	4	106	1.83±0.213	up
2354	P07355	ANXA2	38807.9	7.57	5	223	−1.58±0.128	down
2421	Q6NZI2	PTRF	43449.8	5.51	3	249	2.16±0.241	up
2498	P0C264	SGK110	38862.8	4.71	5	438	−1.69±0.085	down
2500	P63241	EIF5A	17049.5	5.08	2	167	−2.40±0.339	up
2523	P16949	STMN1	17291.9	5.76	2	126	−2.76±0.255	down

Note:

a Protein MW, Protein molecular weight;

b Fold changes (mean ± SD) of U251 cell line vs. U251AR cell line, which were calculated from the DeCyder/spot volume analysis;

c up, up-regulated in the U251AR cell line;

d down, down-regulated in the U251AR cell line.

### Validation of high-expression protein PTRF and VIM in U251AR

**Figure 4:**
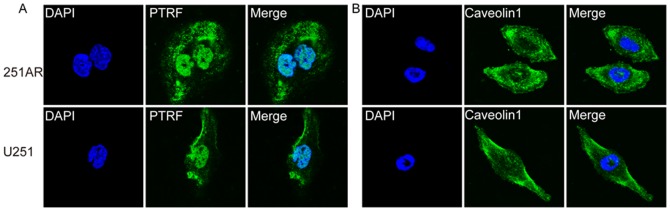
The immunofluorescence staining of PTRF and caveolin1 in U251AR and U251 cells. (A) Immunofluorescence staining of PTRF in U251AR and U251 cells (magnification, 120×). (B) Immunofluorescence staining of caveolin1 in U251AR and U251 cells (magnification, 120×).

To test our proteomic results, we performed Western blot and quantitative RT-PCR to detect the expression of PTRF and VIM. The Western blot and quantitative RT-PCR results confirmed that PTRF and VIM were both highly expressed in U251AR compared with its parental cell U251 (Fig. 3B, 3C,*, p<0.05), which were consistent with our previous results. To gain a comprehensive view of cellular changes induced upon PTRF expression, we used cell immunofluorescence to detect the cellular localization of PTRF and caveolin1 in both U251AR and U251 cells (Fig. 4). PTRF was detected in nucleus and cytoplasm in both cells, with more fluorescence detected in cytoplasm of U251AR than in that of U251. Caveolin1 was also detected in cell membrane and cytoplasm in both cell lines with more fluorescence detected in cytoplasm of U251AR, suggesting that U251AR cells may possess more caveolae than U251 cells.

### Knockdown of PTRF in GBM cell lines increases chemosensitivity

**Figure 5:**
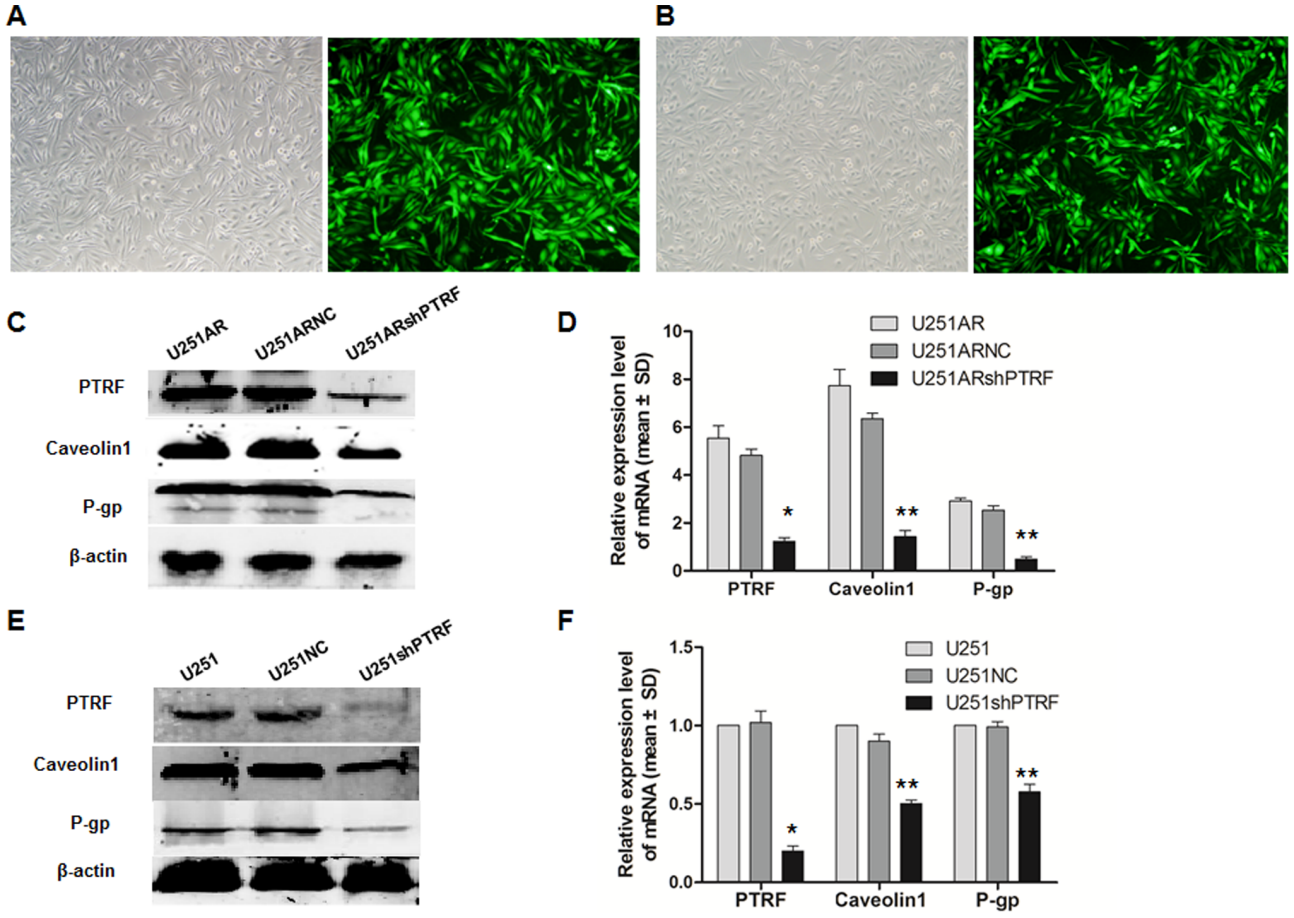
Establishment of the stablely transfected cells and the expression of PTRF in stable transfected cells. (A) Morphology of U251AR cells transfected with shPTRF (U251ARshPTRF cells); (B) Morphology of U251 cells transfected with shPTRF (U251shPTRF cells); Light microscopy, 20× (A, B); Fluorescence microscopy, 20× (A, B); (C, D) The protein and mRNA expression of PTRF, caveoin1, and P-gp in U251AR cells after transfection with shPTRF and negative vector (shNC) by Western bolt and quantitative RT-PCR. (E, F) The protein and mRNA expression of PTRF, caveoin1, and P-gp in U251 cells after transfection with shPTRF and shNC as indicated by Western blot analysis and quantitative RT-PCR.

To further investigate the effect of PTRF on chemoresistance of GBM cells, we knocked down the expression of PTRF using pcDNA6.2-GW/EmGFP-miRNA in both U251 and U251AR cell lines. The morphologies of transfected cells were showed in Fig. 5A and 5B. The interference efficiency was confirmed by Western blotting and quantitative RT-PCR (*, P<0.05, Fig. 5C, 5D, 5E, 5F). Interestingly, silencing PTRF significantly reduced the mRNA and protein levels of caveolin1 and P-gp (**, P<0.05, Fig. 5C, 5D, 5E, 5F).

**Figure 6:**
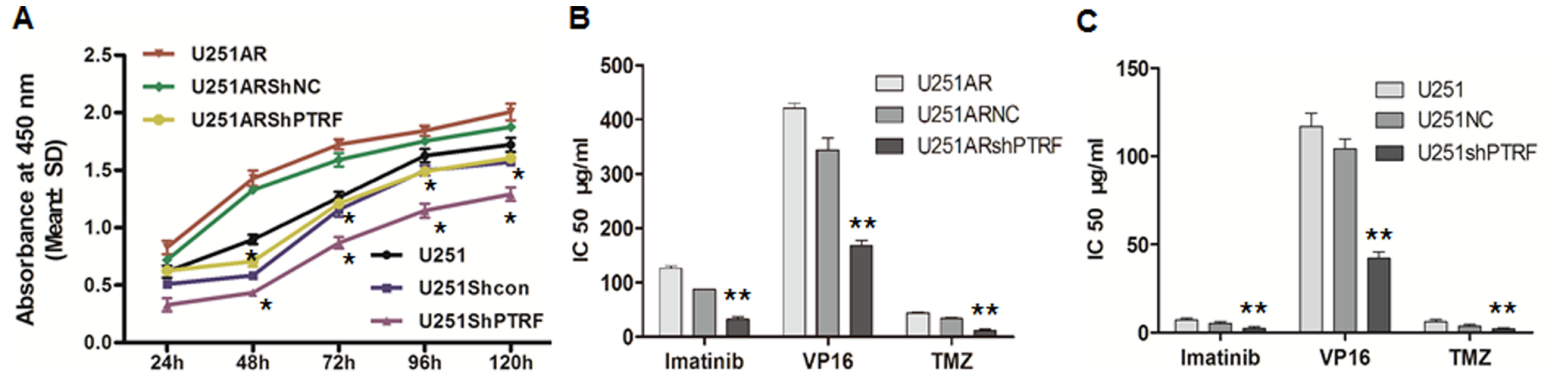
The cell viability (IC50) changes after PTRF konckdown. (A) The cell viabilty of U251AR cells, U251AR cells transfected with shNC and U251AR cells transfected with shPTRF after treatment with 100 µg/mL TMZ for 24 h, 48 h, 72 h, 96 h, and 120 h; (B) The IC50 of U251AR cells and U251AR cells transfected with shNC and shPTRF; (C) The IC50 of U251 cells and U251 cells transfected with shNC and shPTRF. **, P<0.05.

Both PTRF and caveolin1, the two caveolae structure proteins, have been shown to be relevant to chemoresistance. To test the effect of PTRF on cell viability, we treated both cell lines with or without knockdown of PTRF with TMZ (100 µg/mL) for (24 h, 48 h, 72 h, 96 h, and 120 h) and cell viability assay was performed. In this assay, cells with PTRF knockdown showed decreased cell viability when compared with the control cells under the same concentration of TMZ (*, P<0.05, Fig. 6A).

To test the roles of PTRF in GBM chemical drug sensitivities, the IC50 values of U251 cells, U251AR cells and the transfected cells after treatment with imatinib, VP-16, and TMZ were determined by CCK8 assay kit (Fig. 6B, 6C). The IC50 values of shPTRF transfected U251 and U251AR cells after treatment with imatinib, VP-16, and TMZ were significantly decreased by 2.05–3.92 folds (**, P<0.01), indicating that down-regulation of PTRF sensitizes GBM cells to chemotherapeutic drugs.

### PTRF is up-regulated in relapsed GBM specimens and positively correlated with caveolin1

**Figure 7:**
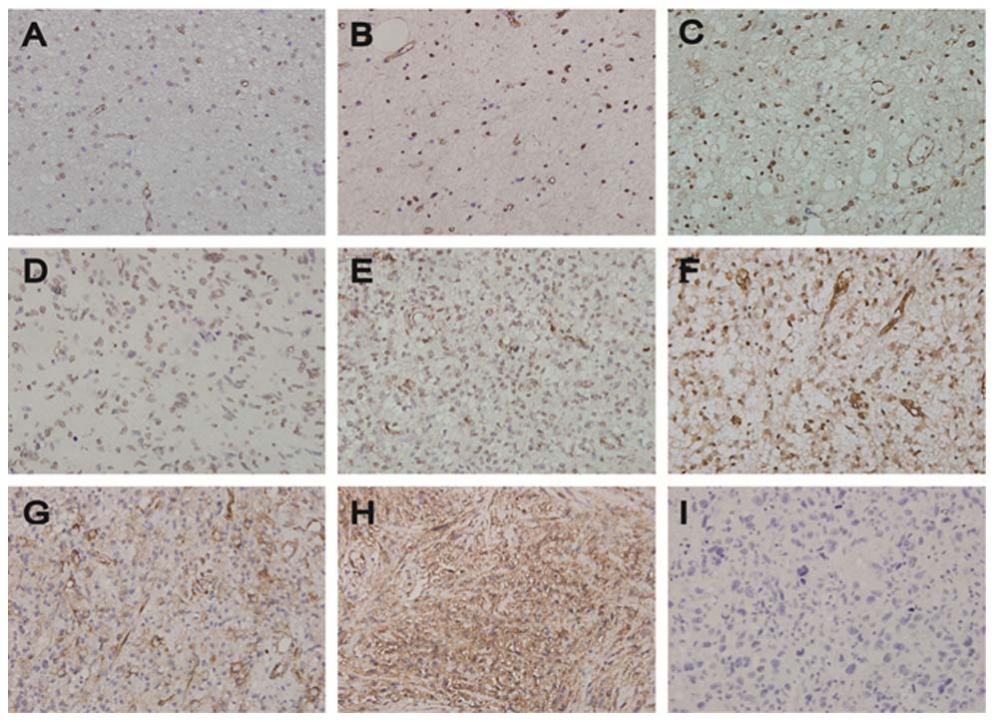
PTRF and caveolin1 expression in astrocytoma tissues as indicated by immunohistochemistry detection. (A) PTRF in normal tissues; (B) PTRF in grade I astrocytoma; (C) PTRF in grade II astrocytoma; (D) PTRF in grade III astrocytoma; (E) PTRF in primary GBM patients; (F) PTRF in relapsed GBM patients; (G) Caveolin1 in primary GBM patients; (H) Caveolin1 in relapsed GBM patients; (I) Negative control.

**Figure 8:**
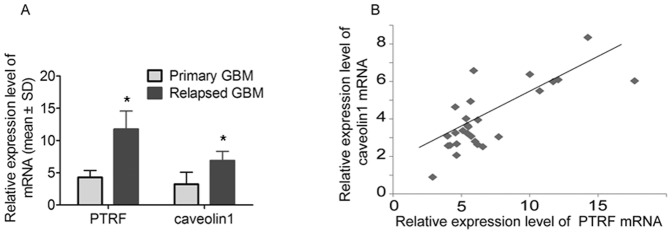
Expression levels of PTRF and caveolin1 in GBM patients. (A) PTRF and Caveolin1 expression in primary GBM and relapsed GBM patients as detected by quantitative RT-PCR (*, P<0.05). (B) The correlation of PTRF and caveolin1 mRNA in the same GBM specimens (2-tailed Pearson correlation, r = 0.766, P<0.01).

In this study, PTRF expression was further detected by immunohistochemistry in tissues from 58 cases of patients with astrocytoma and 6 cases of patients with relapsed GBM. In addition, 8 cases of non-tumor tissues were used as control samples in the immunohistochemistry analysis. The immunohistochemistry assay of PTRF in astrocytoma and normal brain tissue specimens revealed that PTRF was lowly expressed in normal brain tissue and low-grade astrocytoma (grade I and II), but highly expressed in high-grade astrocytoma (grade III and IV, Fig. 7A–7E). We also found that the expression level of PTRF in relapsed GBM patients with treatment of TMZ for 6 months was higher than that in primary GBM patients without treatment of TMZ (Fig. 7E, 7F). Consistent with the expression of PTRF, caveolin1 was also highly expressed in the relapsed GBM patients (Fig. 7G, 7H). A negative control was given in Fig. 7I. Furthermore, the mRNA levels of PTRF and caveolin1 in relapsed GBM patients were significantly higher than those in patients with primary GBM (*, P<0.01, Fig. 8A). PTRF and caveolin1 are two essential components in the biogenesis and function of caveolae. Then, we did correlation analysis between mRNA level of PTRF and caveolin1 in the same GBM specimens. Correlation analysis showed that there was a positive correlation between PTRF mRNA levels and caveolin1 mRNA levels (2-tailed Pearson correlation, r = 0.766, P<0.01, Fig. 8B). All these results indicate that the average expression level of PTRF in the same GBM specimens may be correlated with that of caveolin1.

## Discussion

Although there are some researches on chemoresistance of GBM to TMZ and other chemotherapeutic agents [33], only a few studies used proteomics to investigate chemoresistance of GBM to imatinib. 2D-DIGE is a powerful tool to identify the differentially expressed proteins in different tissues. In this study, we performed 2D-DIGE and MALDI TOF/TOF MS to find proteins that were differentially expressed in GBM cell line U251 and the drug-resistant cell line U251AR. We found 21 MALDI-identified protein spots that showed significant differences both in mRNA expression and in protein expression between the two cell lines, suggesting that imatinib induced differential expression of proteins in U251AR cells.

Among these 21 proteins, VIM and NPM1 have been reported to be associated with cancer chemical drug resistance or GBM chemical drug resistance. VIM showed higher expression level in malignant glioma cells after treatment with a constant concentration of TMZ [34], [35]. NPM1, which played an important role in chemoresistance of tumor cells [36], was also up-regulated in brain tissues of GBM compared to normal tissues [37]. These results suggest that various drug-resistant mechanisms may act together to induce chemoresistance of GBM.

In addition to VIM, PTRF was also identified by immunoblotting analysis using monoclonal antibodies. We analyzed its function in drug resistance, and found that its overexpression contributed significantly to development of imatinib resistance in U251AR cells. PTRF, in the presence of caveolin-1, facilitates formation of caveolae. At a similar expression level, PTRF can induce formation of abundant caveolae [24], [25]. Up-regulated PTRF in chemoresistant breast cancer cell line increases caveolae density [23]. Loss of PTRF expression in prostate cancer and lung cancer is related with cancer progression [21], [22]. PTRF also attenuates the effect of pro-tumor caveolin-1, leading to suppression of tumor growth and metastasis [38].

Caveolin1, a crucial structural protein of caveolae, is also up-regulated in numerous human drug-resistant tumor cells, such as colon adenocarcinoma, breast adenocarcinoma, and lung cancer cells [39]–[42]. Our result showed that the expression of caveolin1 was also up-regulated in U251AR cells. By using immunofluorescence detection, we found that PTRF and caveolin1 were stained more effectively in cytoplasm of U251AR cells, in comparison with those of U251 cells.

PTRF knockdown could decrease the amount of lipid rafts [43] and PTRF is required for distribution of glycosphingolipids into the plasma membrane lipid rafts [23]. Lipid rafts are invaginated to form omega-typed caveolae, which are involved in various cellular events including endocytosis [44], tumorigenesis [45], and MDR [46]. P-gp is enriched in detergent-resistant lipid rafts and associated with caveolin1 in MDR cancer cells [40], [47]. In our study, we knocked down expression of PTRF in U251 and U251AR cell lines, leading to down-regulation of PTRF, caveolin1, and P-gp. The IC50 and cell viability of PTRF silencing cells was significantly decreased when compared with that of the normal cell controls. All these results suggest that PTRF may be associated with drug resistance of GBM cells.

The expression level of PTRF was lower in tumor specimens than that in the normal tissues of non-small cell lung cancer patients [21] and prostate cancer patients [22]. Interestingly, in our study, GBM tissues showed higher PTRF expression levels when compared to the non-tumor and low-grade astrocytoma tissues, suggesting that PTRF was tissue-specific. Caveolin1 was reported to be intensely expressed in tissues of GBM patients compared with the normal brain tissues [28], [29]. We analyzed the correlation between the mRNA levels of PTRF and caveolin1 in patients with primary and relapsed GBMs. Interestingly, the GBM patients with a high PTRF expression tended to exhibit a higher level of caveolin1. Importantly, there was higher PTRF expression level in the relapsed GBM patients than that in the primary GBM patients. The up-regulated PTRF level was in consistent with the higher level of caveolae formation [24]. Therefore, our findings in clinical specimens suggest that PTRF may act as a positive regulator in MDR of GBM patients and that PTRF could modulate the sensitivity of GBM cells to some anticancer drugs. Our results further indicate that PTRF may be used as a novel biomarker of GBM chemoresistance and as a potential target for treatment of GBM. However, the exact mechanism underlying the role of PTRF in chemoresistance of GBM cells still needs further investigation.

In summary, using proteomics methods, we showed that chemoresistance of GBM was related with many factors. Among these factors, PTRF may play important roles in drug resistance of GBM. In addition, we found that PTRF expression was up-regulated in GBM specimens and expressed at higher levels in the relapsed GBM patients. Therefore, PTRF may serve as potential biomarkers for early diagnosis and prognosis of GBM, and as potential therapeutic targets of GBM.
